# Worsening Preseptal Cellulitis With an Orbital Abscess and Intracranial Extension in a Pediatric Patient

**DOI:** 10.7759/cureus.73772

**Published:** 2024-11-15

**Authors:** Taner B Celebi, Ahmad Shamulzai, Hadi Dahhan

**Affiliations:** 1 Family Medicine, Northwell Health, Plainview, USA; 2 Neurology, Northwell Health, Manhasset, USA; 3 Medicine, Plainview Hospital, Plainview, USA

**Keywords:** ct scan head, mri head, orbital, orbital cellulitis, pediatric head injury, preseptal orbital cellulitis

## Abstract

This case presents a 12-year-old male patient diagnosed with preseptal cellulitis that progressed to a subperiosteal orbital abscess and eventually intracranial extension, despite outpatient antibiotic therapy. Initially treated with oral antibiotics for left eyelid swelling and pain, his condition worsened, prompting hospital admission and eventual surgical intervention. Imaging revealed multiple abscesses and a hematoma, causing mass effect on the globe and extraocular muscles. Despite aggressive medical management, surgical drainage was required, including a craniotomy for drainage of an epidural abscess. This case highlights the importance of timely escalation of care when complications arise from preseptal cellulitis.

## Introduction

Preseptal cellulitis is an inflammation of the soft tissues of the eyelid anterior to the orbital septum, typically revolving around an infectious process requiring oral antibiotics. Periorbital cellulitis is most common in children and is primarily caused by sinusitis or trauma [1]. Sinusitis due to Haemophilus influenzae type B (HIB) was the cause of most infections with bacteremia prior to the HIB vaccine, with now only 3.5% of cases in the current era [2]. Presently, the most common causative organisms in orbital cellulitis in the current era include gram-positive cocci [3], typically Staphylococcus aureus followed by Streptococcus species [4]. On the other hand, trauma-induced infections had a male prevalence in low- and middle-income countries, possibly due to the higher propensity for work-related injuries in these regions [5].

Initially a superficial infection, preseptal cellulitis can progress to orbital cellulitis, thereby becoming a serious vision-threatening disease process. Preseptal cellulitis typically presents with unilateral eyelid swelling and edema [6]. In contrast, patients with orbital cellulitis present with similar findings plus ocular symptoms such as proptosis, eye pain, decreased vision, and limited extraocular motility [6]. This progression of infection deeper into the ocular globe comes with the increased risk of abscess formation with sequelae intracranial complications often necessitating surgical intervention.

With the threatening progression from preseptal to orbital cellulitis, it becomes imperative to differentiate the pathological progression of such an infection. This report details a case of preseptal cellulitis in a 12-year-old male patient, complicated by orbital abscess formation and intracranial extension, requiring surgical drainage and prolonged antibiotic therapy.

## Case presentation

A 12-year-old male patient with a history of a pelvic kidney presented with left upper eyelid swelling and pain, worsening over two weeks. His symptoms started after a wrestling match, where he sustained an injury to his left eye without visible trauma. Several days later, swelling and discomfort ensued, and his primary care physician diagnosed him with preseptal cellulitis. He was treated with amoxicillin 500 mg every eight hours and clindamycin 300 mg every eight hours. Despite compliance with the antibiotics, his condition deteriorated, with increased pain and swelling, especially during eye movements.

The patient returned to the ED after six days of antibiotic therapy with worsening symptoms, including left eye swelling, inability to open the eye, significant pain, and mild headache. He reported no vision changes but had difficulty moving his left eye, especially when looking to the right.

On examination, the patient was well-nourished but in moderate distress. Vital signs were within normal limits. The temperature of the patient was 37.4°C. The heart rate and blood pressure were 86 bpm and 99/57 mmHg, respectively. The respiratory rate was 20 breaths per minute, with oxygen saturation at 98% on room air. 

The left upper eyelid was swollen, erythematous, and tender to palpation. Extraocular movements in the left eye were limited, with pain on right gaze. Visual acuity in the left eye was slightly diminished (20/25), while the right eye remained normal at 20/20.

**Figure 1:**
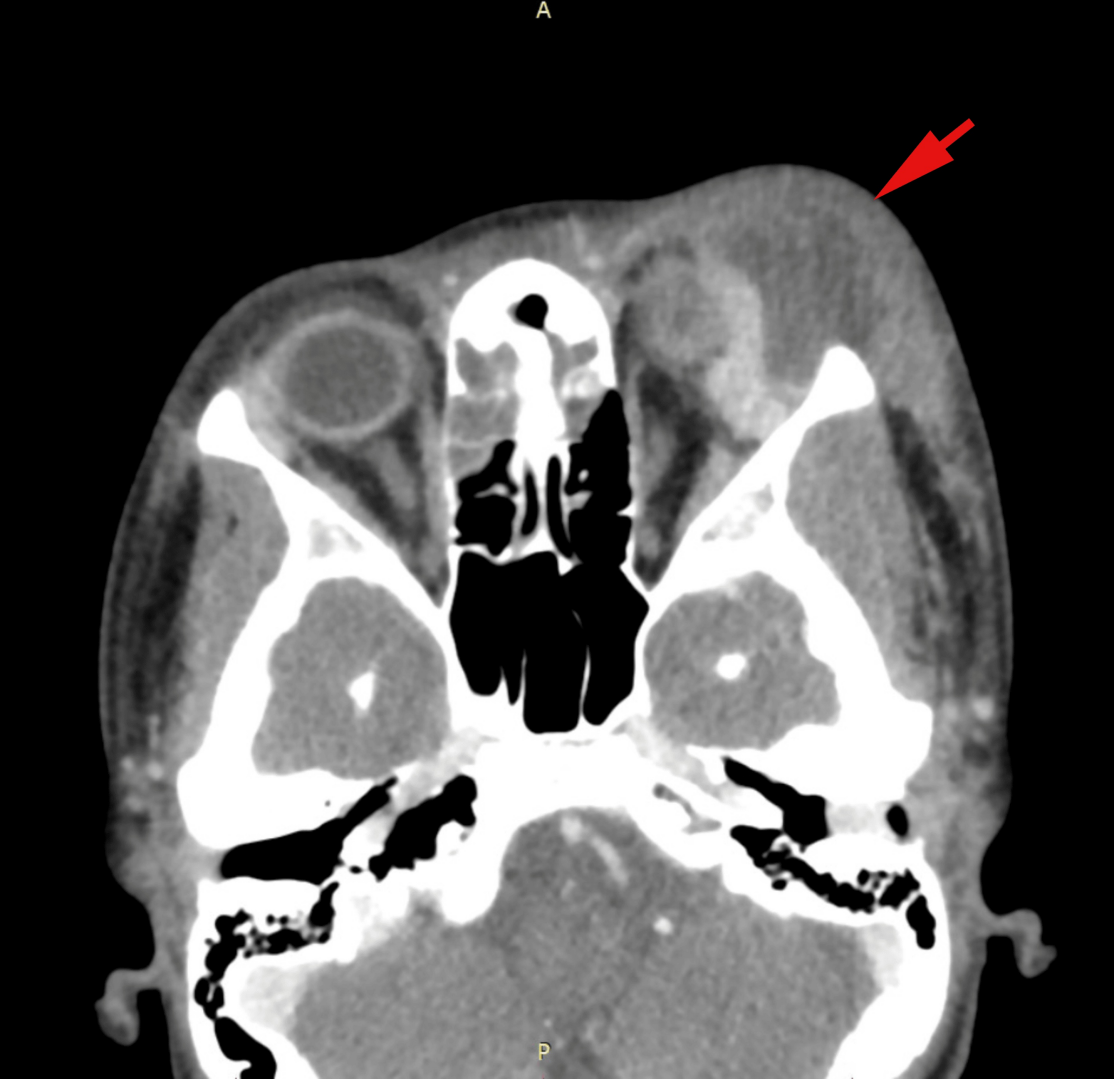
CT of left orbital and frontal subperiosteal abscesses Axial contrast-enhanced CT scan demonstrating a 3.1 x 2.6 x 2.5 cm subperiosteal abscess in the left orbit, causing mass effect on the globe, superior rectus, and lacrimal gland. A secondary abscess (1.7 x 0.7 x 1.9 cm) is noted along the left frontal bone. Marked periorbital edema and near-total opacification of the frontal and ethmoid sinuses are evident.

A contrast-enhanced CT (Figure 1) scan of the orbits revealed worsening preseptal cellulitis and the formation of two subperiosteal abscesses, the largest measuring 3.1 x 2.6 cm, located along the superior and lateral orbital walls, causing mass effect on the globe and extraocular muscles. A smaller abscess, measuring 1.7 x 1.9 cm, was also present. No evidence of osseous erosion or intracranial involvement was noted.

The patient was admitted for IV antibiotic therapy with ceftriaxone 2 g IV every 24 hours and vancomycin 915 mg IV every eight hours for possible methicillin-resistant Staphylococcus aureus (MRSA) coverage. Despite this treatment, his symptoms continued to worsen over the next 48 hours, and repeat ophthalmologic evaluation confirmed increased swelling and conjunctival injection. Ophthalmology and ENT teams recommended surgical drainage, which was performed under general anesthesia.

During surgery, two subperiosteal abscesses were drained, and intraoperative cultures were sent for microbiological analysis. The patient’s antibiotic regimen was adjusted postoperatively to piperacillin-tazobactam 80 mg/kg IV every eight hours, and vancomycin was continued. This upgrade in antibiotics was secondary to worsening symptoms on the previous regimen. Initial cultures were negative, and the patient’s symptoms gradually improved.

**Figure 2:**
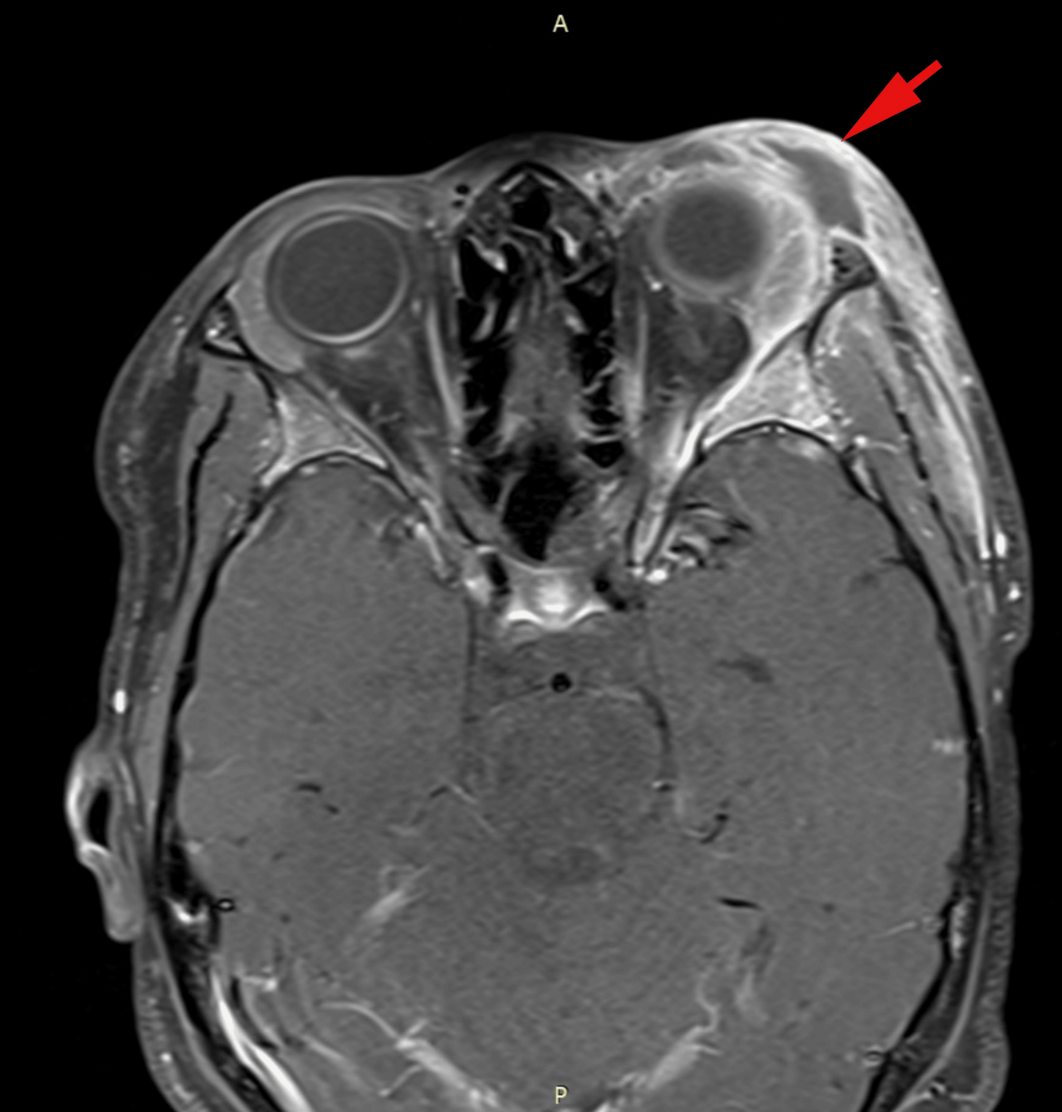
Post-contrast axial T1 fat-suppressed MRI of the orbits Axial T1 fat-suppressed post-contrast MRI showing a left frontal epidural abscess with anterior midline involvement and increased signal intensity. The abscess measures approximately 1.7 cm x 0.9 cm in the transverse plane and 3.8 cm in the cephalocaudal plane, causing posterior displacement of the superior sagittal sinus. Dural thickening and enhancement in the anterior frontal region are noted, consistent with pachymeningitis. Left orbital abscesses in the superior eyelid and postseptal extraconal space are visible, along with periorbital soft tissue swelling and slight left-sided proptosis.

**Figure 3:**
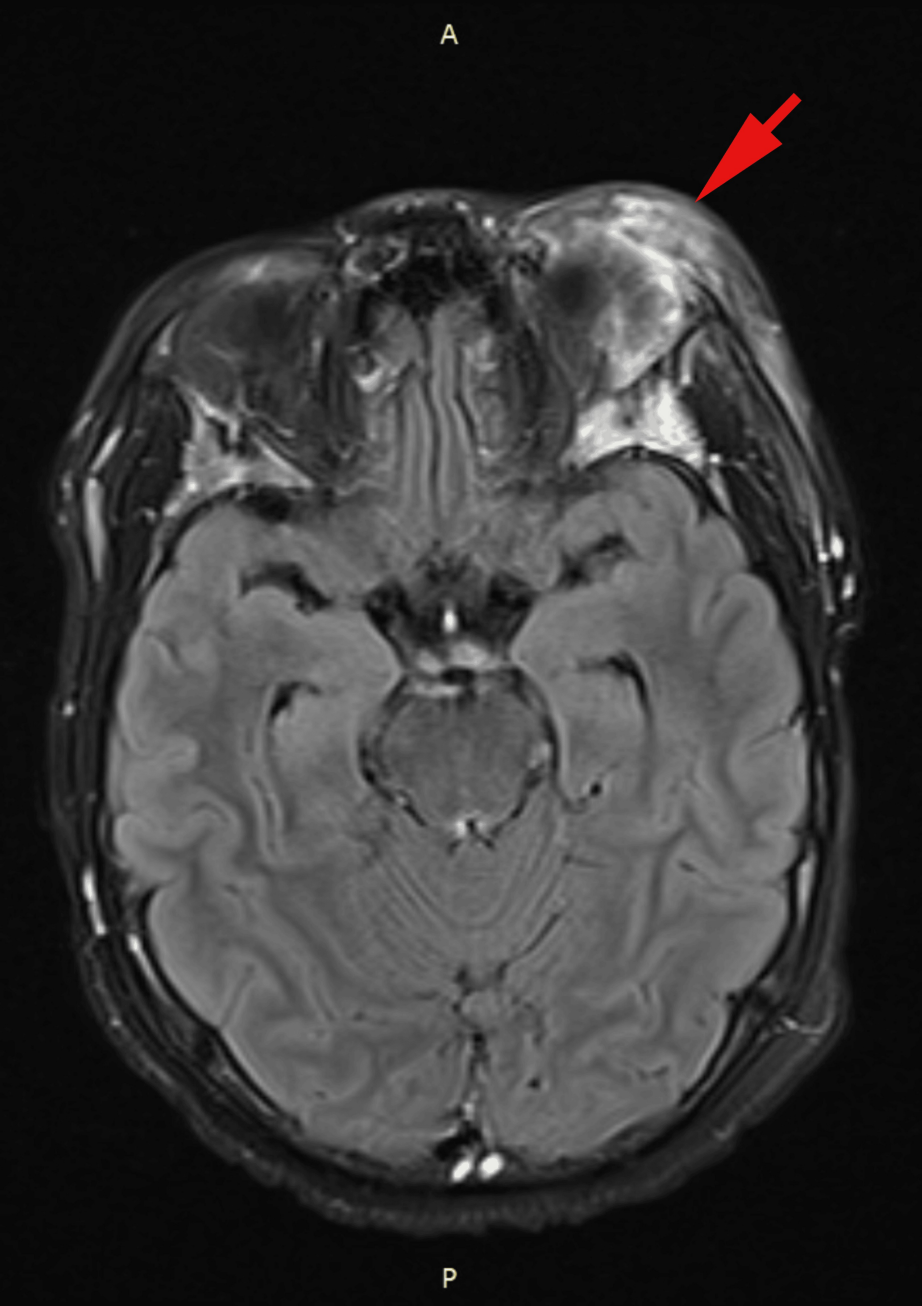
Post-contrast Axial T2 fluid-attenuated inversion recovery (FLAIR) MRI Axial T2 FLAIR post-contrast MRI demonstrating extensive left-sided preseptal and postseptal orbital abscesses with associated dacryoadenitis of the left lacrimal gland and diffuse infectious phlegmon extending across periorbital tissues. Bone marrow edema in the frontal calvarium and greater sphenoid wings is evident, suggesting osteomyelitis. Right maxillary sinus opacification indicative of sinusitis is also present.

However, on day six of hospitalization, the patient developed a left-sided intention tremor, with a mild pronator drift on the left side. An MRI of the brain (Figures 2, 3) showed an epidural abscess, likely due to a periorbital spread of infection. Neurosurgery was consulted, and a craniotomy with supratentorial abscess drainage was performed. The procedure involved a frontal craniectomy for the evacuation of the epidural abscess, with the placement of an epidural Jackson-Pratt (JP) drain. Postoperatively, the incision was closed with monocryl sutures, and the patient recovered well.

A peripherally inserted central catheter (PICC) was placed for long-term antibiotic administration. His antibiotic regimen was switched to IV ceftriaxone, by mouth (PO) linezolid 600 mg twice daily (status post seven days total of vancomycin), and PO metronidazole 500 mg every eight hours.

Postoperative course

Following the craniotomy and abscess drainage, the patient’s clinical status improved significantly. On postoperative day three, his left eye could be partially opened, and visual acuity remained stable at 20/25. His extraocular movements improved, though some discomfort persisted with lateral gaze. A follow-up MRI showed a reduction in the size of the abscesses, with no further intracranial complications. The patient was noted to have mild mechanical ptosis but was otherwise stable.

The patient was discharged home with a continued four-week course of IV ceftriaxone 2 g every 12 hours via his PICC line, accompanied by oral linezolid 600 mg twice daily and oral metronidazole 500 mg every eight hours. He was scheduled for follow-up with infectious disease in one week, and the tentative duration of antibiotics was set for four weeks, depending on outpatient progress.

## Discussion

This case illustrates the potential severity of preseptal cellulitis, which can rapidly escalate into a more serious infection involving the orbit and even intracranial structures. The eye is housed within the bony orbit, lined by a protective fibrous membrane (periosteum) that serves as the only true barrier between the orbit and the delicate, thin-walled ethmoid sinus [7]. Consequently, ethmoid sinusitis is the typical trigger for orbital cellulitis in children under seven years old, as it is the only fully developed sinus at birth [8]. With the development of sinusitis, bacteria invade through the thin medial wall of the orbit featuring numerous perforations for blood vessels, nerves, and natural fenestrations, thereby allowing bacteria to travel retrogradely via the venous system, further complicating the infection [9].

Pediatric patients are particularly susceptible to complications such as orbital cellulitis and subperiosteal abscesses due to the close anatomical relationship between the sinuses, orbit, and intracranial space. Infections can directly involve the optic nerve by compressing the orbital compartment resulting in visual impairments, altered color vision, or a relative afferent pupillary defect [10]. Infection can progress further, consolidating into an abscess, increasing its complexity.

Subperiosteal abscesses develop when infection localizes between the periorbita and the orbital bones [9]. Such infections can continue to worsen with the extension of the abscess beyond the subperiosteal space into the orbit itself. With the compromised orbit, the absence of valves in the venous drainage system of the eye exacerbates the infection by permitting the infection to progress from the orbit to the cavernous sinus or brain, potentially leading to meningitis or intracranial abscesses [11].

This case shows the progression of preseptal cellulitis into an epidural abscess, requiring a coordinated multidisciplinary approach involving ophthalmology, ENT, and neurosurgery teams for surgical drainage. Early detection of orbital and intracranial involvement through imaging was crucial in determining the need for surgical intervention. Despite initial treatment with broad-spectrum outpatient antibiotics, the patient ultimately required IV antibiotics, surgical drainage, and a craniotomy for epidural abscess evacuation. This case highlights the critical importance of timely recognition, imaging, and surgical management to prevent severe complications, including vision loss, neurological deficits, or life-threatening intracranial infections.

## Conclusions

Preseptal cellulitis can evolve into a severe infection if not promptly and adequately treated. In pediatric patients, an early recognition of orbital and intracranial complications through imaging is vital. Surgical intervention, as seen in this case, can prevent catastrophic outcomes. This case emphasizes the importance of a multidisciplinary approach and a timely escalation of care in complicated cases of preseptal cellulitis. In this patient, a four-week course of IV ceftriaxone via a PICC line was required to ensure the complete resolution of the infection.

## References

[1] Ekhlassi T, Becker N (2017). Preseptal and orbital cellulitis. Dis Mon.

[2] Ambati BK, Ambati J, Azar N, Stratton L, Schmidt EV (2000). Periorbital and orbital cellulitis before and after the advent of Haemophilus influenzae type B vaccination. Ophthalmology.

[3] Harris GJ (1994). Subperiosteal abscess of the orbit. Age as a factor in the bacteriology and response to treatment. Ophthalmology.

[4] Santos JC, Pinto S, Ferreira S, Maia C, Alves S, da Silva V (2019). Pediatric preseptal and orbital cellulitis: a 10-year experience. Int J Pediatr Otorhinolaryngol.

[5] Al-Madani MV, Khatatbeh AE, Rawashdeh RZ, Al-Khtoum NF, Shawagfeh NR (2013). The prevalence of orbital complications among children and adults with acute rhinosinusitis. Braz J Otorhinolaryngol.

[6] Bae C, Bourget D (2024). Periorbital cellulitis. https://www.ncbi.nlm.nih.gov/books/NBK470408/.

[7] Tsirouki T, Dastiridou AI, Ibánez Flores N, Cerpa JC, Moschos MM, Brazitikos P, Androudi S (2018). Orbital cellulitis. Surv Ophthalmol.

[8] Gordon AA, Phelps PO (2020). Management of preseptal and orbital cellulitis for the primary care physician. Dis Mon.

[9] Hornblass A, Herschorn BJ, Stern K, Grimes C (1984). Orbital abscess. Surv Ophthalmol.

[10] Lima V, Burt B, Leibovitch I, Prabhakaran V, Goldberg RA, Selva D (2009). Orbital compartment syndrome: the ophthalmic surgical emergency. Surv Ophthalmol.

[11] Frank GS, Smith JM, Davies BW, Mirsky DM, Hink EM, Durairaj VD (2015). Ophthalmic manifestations and outcomes after cavernous sinus thrombosis in children. J AAPOS.

